# Ocular features in sepsis - a scoping review

**DOI:** 10.1186/s12886-026-04647-6

**Published:** 2026-02-07

**Authors:** Yatharth Datta, Sruthi Vijendran, Yogish Subraya Kamath

**Affiliations:** Department of Ophthalmology, Kasturba Medical College, Manipal Academy of Higher Education, Manipal, Karnataka 576104 India

**Keywords:** Sepsis, Ocular disease, Ocular inflammation

## Abstract

**Objective:**

This review aimed to provide a comprehensive overview of the types of ocular manifestations observed in patients suffering from systemic sepsis including modifications in management of sepsis with ocular involvement.

**Introduction:**

Sepsis is a life-threatening condition resulting in widespread organ dysfunction, driven by dysregulated host responses to infection. In patients with altered sensorium or impaired communication, timely detection of ocular involvement becomes particularly challenging. Delays in recognition can lead to irreversible visual loss, underscoring the need for early ophthalmic evaluation in this population.

**Inclusion Criteria:**

This review included adult patients (aged > 18 years) from a global population who exhibited ocular manifestations in the context of systemic sepsis, defined by Sepsis 2 and Sepsis 3 criteria. Studies focusing solely on pediatric patients, non-septic conditions, or without ocular features were excluded.

**Methods:**

A comprehensive search was conducted on December 29, 2025, across four electronic databases—PubMed (MEDLINE), Embase (Elsevier), Scopus (Elsevier), and ProQuest. Boolean operators “OR” and “AND” were applied to combine synonyms and cross-domains related to sepsis and eye disease. Studies published in English from the year 2000 onward were included. Exclusion criteria encompassed non-English articles, reviews, conference abstracts, non-sepsis-related conditions, and non-human or cell-based studies.

**Results:**

Of 2276 articles screened, 22 met the inclusion criteria. These comprised case reports, case series, and observational studies from diverse global settings. Fungal infections, particularly Candida albicans, emerged as the leading cause of ocular involvement, followed by Staphylococcus aureus and Klebsiella pneumoniae. Posterior segment findings such as chorioretinitis and vitritis were most frequently reported. Anterior segment and periocular signs were infrequently reported in studies.

**Conclusions:**

Ocular manifestations constitute a serious complication in sepsis, with visual impairment and posterior segment involvement commonly originating from bacterial and fungal infections, often stemming from the urinary tract. Early detection in sepsis patients with positive blood cultures, combining ocular and systemic interventions, can improve outcomes.

**Supplementary Information:**

The online version contains supplementary material available at 10.1186/s12886-026-04647-6.

## Introduction

**Keywords**: sepsis, ocular disease, ocular inflammation

The eye can be severely affected by a systemic focus in patients suffering from sepsis. Often, these are severely vision-threatening [1]. Hence, urgent ophthalmological inspection in sepsis becomes important. Conscious and verbal patients might be able to draw attention to any visual morbidity that they may experience. This becomes difficult in seriously ill patients, who are in altered states of consciousness, who are at risk of late detection and a higher rate of complications. Sepsis is defined as life-threatening organ dysfunction caused by a dysregulated host response to infection. Organ dysfunction can be detected as an acute change in total Sequential Organ Failure Assessment (SOFA) score ≥two points consequent to the infection. Patients who are at risk of organ damage have a risk of prolonged hospitalization, ICU admission and an increased risk of death due to the infection. Often, through objective indicators, it is possible to detect ocular features of infections in those with systemic foci of infection [2]. The detection of these infections can improve the prognosis if detected early [3]. A 2020 estimate of global blindness states that 49 million are blind and 300 million suffer from moderate to severe visual impairment [4], but there is a lack of prevalence-based data attributing systemic sepsis to ocular blindness. A 2020 systematic review by Breazzano et al. [5] reported on the value of ocular screening in patients with candidemia. Recent work reports changes in blood flow in conjunctival and retinal microvasculature in patients with sepsis [6], which also reflected cerebral microvascular changes in critical illness. By including various microbiological and systemic causes of sepsis, the scoping review aimed to describe sepsis etiologies and ophthalmological manifestations and report on the frequency of occurrence of ocular conditions within the studies. Secondly, changes in management of systemic sepsis, with the presence of ocular involvement including visual morbidity, is reported. This can assist healthcare providers in clinical practice by providing risk-stratified evidence rather than relying solely on pathogen-based screening.

## Methodology

We adopted a scoping review methodology to provide a broad overview of the ocular features in patients suffering from sepsis, guided by a protocol. The “Preferred Reporting Items for Systematic Reviews and Meta-Analyses (PRISMA) Extension for Scoping Reviews Checklist” [7] was utilized as an aid to report this review (Supplementary Table 1). The “Arksey and O’ Malley” Framework was utilized for the detailed reporting of the review [8].

## Research question

What are the ocular features in patients with sepsis?

We used the population, concept, context design criteria for identification of the studies.

## Population

We included the global adult population above 18 years with sepsis, with a documented source of infection. Participants with pediatric sepsis, non-human studies and cell-based studies, studies on dead tissue were excluded.

## Concept

To provide a comprehensive overview, this review included articles describing the manifestations of sepsis in the eye in adults, with ocular and systemic foci, and related causes of infection. Studies consisting of patients with diagnosed sepsis detected using standard methods (clinical or blood cultures) having ophthalmological manifestations that were confirmed to be due to the sepsis or with the most likely cause as sepsis, were considered eligible for the review. Local inflammatory and neoplastic pathologies, as well as metastatic tumor emboli, and the local spread of inflammation from surrounding ocular tissues with no infectious foci of inflammation, studies mentioning the eye as a source of infection, and articles describing drug toxicity were excluded. Purely mechanistic or pathophysiological findings of studies were excluded. Those describing solely sepsis or ocular features without linking ocular features with systemic sepsis were excluded.

## Context

Patients from hospitals or intensive care clinics, along with their documented outcomes and follow-up, were described in detail, focusing on ocular complications and resultant morbidity and mortality, regardless of geographical boundaries. Community cases, patients treated on an outpatient basis, with sepsis-related ocular conditions were excluded.

### Study designs (eligibility criteria)

We included studies describing ocular manifestations in systemic sepsis, identifying vision abnormalities, anatomical site of involvement (anterior segment, posterior segment, or periocular), and clinical manifestations (e.g., conjunctivitis, keratitis, uveitis, cataract, vitritis, chorioretinitis, retinal disease, optic nerve involvement) and reporting the type of infective organism (bacterial, fungal, or mixed). We included studies establishing a causal association between ocular diseases and systemic sepsis. If the study reported worsening of pre-existing ocular diseases with sepsis, such as glaucoma, retinopathy due to other systemic comorbidities such as diabetes and hypertension, the reported finding was excluded. Eligible study designs included case reports, case series, retrospective and prospective observational studies, cohort studies, clinical trials, and general chart reviews.

We excluded qualitative studies, systematic and narrative reviews, study protocols, editorials, letters to the editor, conference abstracts, and grey literature. Non-human research, cell-line and simulation studies, genetic and epigenetic studies, book chapters, news articles, and studies without English full-text access were also excluded.

### Identification of the relevant literature

The search strategy was composed by a reviewer trained in evidence synthesis (S.V.) after identifying relevant search terms from the “Medical Subject Headings” (MeSH) library. Keywords used in relevant articles were identified through a preliminary search with term harvesting conducted on PubMed to develop the search strategy, utilizing both indexed and free-text terms of “eye” and “sepsis”. A discussion with subject experts was conducted to assess the relevance of the search strategy. To conduct a comprehensive search, four electronic databases of PubMed (MEDLINE), Embase (Elsevier), Scopus (Elsevier), and ProQuest were systematically searched on 29 December 2025 by S.V. and Y.D. independently and validated by Y.K. An online polyglot translator was used to aid the translation of the PubMed search strategy to the remaining databases [9]. Studies published in peer-reviewed journals from 2000 to 29 December 2025 were included. Validation of the search strategy was done using the Peer Review of Electronic Search Strategies (PRESS) checklist by an independent search expert outside the review team [10]. The search strategy has been attached in Supplementary Table 2.

### Selection of studies

The results of the search were exported to Rayyan.Ai software. After conducting a calibration exercise among the reviewers, following duplicate removal, the screening of titles and abstracts was carried out through two independent reviewers (Y.D. and S.V.). The screening of the full text was done by two reviewers (Y.D. and S.V.). Any conflicts that arose in this process were resolved upon discussion with an independent reviewer (Y.K).

### Data charting

Zotero was used as the citation manager. Two independent reviewers conducted the charting of data independently (S.V., Y.D.). The adequacy of reporting in the chosen studies was cross verified by three reviewers (S.V., Y.D., Y.K.). Reporting on bias within the studies was not done. The parameters extracted were publication details, setting, study design and objective, sample size, methodology, results including vision, ocular involvement features, diagnostic modality, complications, sources of sepsis and treatment provided, including other findings observed, by using a self-developed calibrated proforma on Microsoft Excel 360.

### Collecting, summarizing and reporting results

To avoid data overlap, a deduplication strategy was applied at the title and abstract stage with Rayyan.ai software, and during full-text review, possible overlaps were prevented after data extraction upon discussion with the third reviewer (Y.K.). Studies were coded by author names as identifiers. Numerical data about patients studied, frequency of ocular conditions and subclassification depending on the anatomical ocular region involvement were tabulated.

Microbiological etiology were further categorized as bacterial, fungal and other infectious etiology. Source of infection, including systemic organ involved and initial foci of infection, including relevant associations between ocular conditions and sepsis, including management of sepsis with ocular involvement, were charted in separate tables following independent charting and calibration. A narrative approach was adopted to summarize the findings.

### Stakeholder consultation

This step was not performed due to limited access to intensive care stakeholders, with time constraints in scheduling appointments.

## Results

**Fig. 1:**
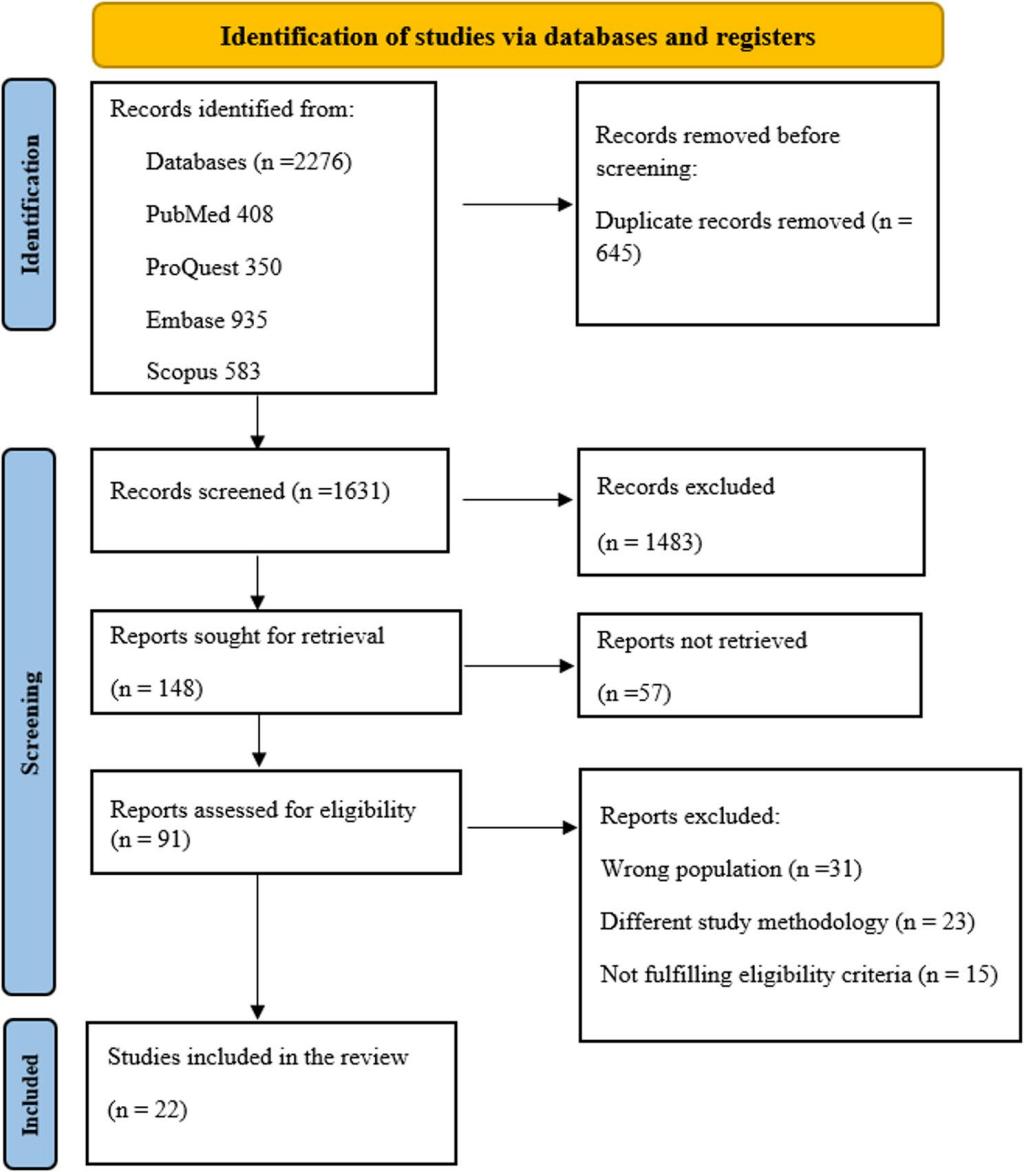
PRISMA 2020 flow diagram for scoping reviews which included searches of databade ocular features in sepsis. Source: page MJ, et al. BMJ 2021;372:n71. doi: 10.1136/bmj.N71. This work is licensed under CC BY 4.0. To view a copy of this license, visit https://creativecommons.org/licenses/by/4.0/

A search on the electronic databases of PubMed (Medline NCBI), Embase (Elsevier), Scopus (Elsevier), and ProQuest (Clarivate) was conducted on 29 December 2025, which revealed a total of 2276 articles. Following duplicate removal, 645 articles were removed with the Rayyan.ai software. Subsequently, 1631 articles were processed for title and abstract screening. After the removal of 1483 articles, 148 articles were retrieved for full-text screening. As the full texts of 57 did not meet the eligibility criteria, 91 articles were screened. Of these, 69 articles did not meet the eligibility criteria for the reasons described, and 22 articles were chosen for the comprehensive review. The PRISMA flow chart represents the stepwise screening process in Fig. 1.

## Characteristics of included studies

### Study settings

Among the included 22 studies, five studies were conducted in United States of America (USA), three studies in India, two in Malaysia and two in Australia, one study each was done in Canada, Denmark, Italy, Japan, Lithuania, South Korea, Taiwan, Thailand, Turkey and in United Kingdom respectively.

### Study designs

The data required to elaborate on the ocular features in sepsis were extracted from ten case reports (45.5%), six retrospective studies (27.3%), three prospective observational studies (13.6%), two cohort studies (9.1%), and one case series (4.5%). The general characteristics of the individual studies are reported in Supplementary Table 3

### Ocular features in sepsis

The studies included in this review painted a diverse, yet compelling picture of how systemic sepsis can manifest in the eye. A total of 1526 people were included across the studies. While the overall data were heterogeneous, some consistent patterns emerged regarding the causative organisms, the systemic sources of infection, ocular involvement, and the management of sepsis with ocular manifestations.

### Microbial etiology and systemic foci

A total of 10 (45.45%) studies report fungal infections as their microbial etiology; the majority of studies (9; 40.91%) identified Candida species, particularly Candida albicans, as the cause of infection [11–19]. One study(4.55%) reported of Scedosporium [20] as the source of infection. In studies by Huynh et al. [12] and Kirkegaard et al. [13], roughly one in six patients with candidemia developed ocular signs such as vitritis or chorioretinitis. These cases were frequently diagnosed in the context of bloodstream infections, with catheter-related seeding being a common source.

A total of 14(63.64%) studies report bacterial infections as their microbial etiology. [11, 14, 17, 21–31] On the bacterial spectrum, Staphylococcus aureus and Pseudomonas aeruginosa were implicated in 5(22.73%) [11, 14, 21, 22, 26] and 2 studies(9.09%) [14, 22] respectively. In a large Japanese cohort studied by Nagasako et al., about 8% of patients with bacteremia exhibited retinal changes such as cotton wool spots or intraretinal hemorrhages [11]. Geographic variation in microbial isolates was evident: gram-negative infections, especially Klebsiella pneumoniae, noted in 4(18.18%) studies, were more commonly reported in India and Southeast Asia [14, 23, 25, 31], whereas gram-positive organisms dominated reports from high-income countries [11, 13, 15, 18, 20, 28].

With respect to the systemic focus, the urinary tract was identified as the primary sites of infection in 7 studies(31.82%) [6, 11, 14, 17–19, 29]. Central venous catheter was identified in 5 (22.73%) studies [11, 12, 14, 17, 20]. Other systemic origins included the soft tissue [6, 11, 14], and the gastrointestinal system [6, 23, 31]; infective endocarditis in 2(9.09%) studies [11, 26], nasal wall abscess [21] and dental infections [28]. However, 5 studies(22.73%) [13, 15, 16, 19, 24] did not explicitly define the source of infection. This has been summarized in Table 1.

**Table 1:** Organism isolated and source of infection among included studies (*N* = 22)

Author, Journal, Study Year Country	Organism isolated (if reported)	Source of infection
Nagasako et al. [11]	Bacterial: Staphylococcus aureus (most common). Fungal: Candida albicans (most common). Candida glabrata.	Catheter-related bloodstream infections. Infective endocarditis. Urinary tract infections. Soft-tissue and bone diseases.
Simkiene [32],	NR	Pneumonia (9 patients), Urinary tract infection (3 cases), one case of Skin and soft tissue infection, 16 cases of Peritonitis, and 11 cases of other infections.
Huynh et al. [12]	Candida	Risk factors identified were: Intravenous catheter (19 patients; 53%), malignancy (13 patients; 36%), abdominal surgery within 1 month (10 patients; 28%), diabetes (6 patients; 17%), total parenteral nutrition (9 patients; 25%), hemodialysis (3 patients; 8%), and systemic steroid use (2 patients; 6%)
Kirkegaard et al. [13]	Candida	NR
Santanaraman et al. [14]	Bacterial in four cases (Staphylococcus aureus in two, Pseudomonas aeruginosa in one, and Klebsiella pneumoniae in one) and fungal in one (Candida albicans).	Central venous Catheter-related bloodstream infections (two patients), urosepsis (two patients), and subcutaneous infection (one patient).
Mohammad et al. [19]	Candida albicans and Candida glabrata	NR
Gluck et al. [15]	Candida species, Candida albicans being the most frequent.	NR
Yannis et al. [16]	Candida albicans (2), Candida tropicalis (2), Aspergillus, Candida kefyr, and non-candidal yeast	NR
Ilhami et al. [22]	Pseudomonas aeruginosa (27.3%) and Staphylococcus aureus (22.7%).	hospital-acquired
Rodríguez-Adrián et al.[17]	Candida and bacterial sepsis	Catheter (central venous or urinary)
Chen et al. [23]	Klebsiella pneumoniae	liver abscess in the majority of cases.
Yaisawang et al. [24]	Burkholderia pseudomallei	NR
Ismail et al. [25]	Klebsiella pneumoniae	Urinary Tract Infection (UTI) And blood culture
Amir et al. [18]	Candida	UTI
Krista et al. [26]	Methicillin-sensitive Staphylococcus aureus.	Bacterial endocarditis
Saidi et al. [21]	Methicillin-Sensitive Staphylococcus Aureus (MSSA)	nasal wall abscess
Suchit et al. [27]	Salmonella typhi	Isolated from blood -Enteric fever
Allegrini et al. [28]	Streptococcus constellatus, a commensal organism of the oral mucosa.	Dental infection (maxillary abscess).
Padmaja et al. [29]	Bacteriuria ( > 105 colony forming units of Escherichia coli per mL)	UTI (urine culture)
Karinya et al. [30]	Meningococcus	multi-organ failure and DIC.
Chang et al. [31]	Klebsiella pneumonia	Liver abscess
Penelope et al. [20]	Scedosporium apiospermum Scedosporium prolificans	Central venous catheter, blood cultures

DIC: Disseminated Intravascular Coagulation, MSSA: Methicillin-Sensitive Staphylococcus Aureus, NR: Not reported, UTI: Urinary Tract Infection

## Spectrum of ocular involvement

### Visual impairment

A total of 15 (68.18%) studies reported on decreased vision with sepsis. Majority of studies (12; 54.54%) described greater visual loss [11, 14, 16, 18, 20, 21, 24, 26, 27, 29–31] and three(13.64%) studies described mild visual loss [12, 23, 25]. Two studies(9.09%) reported that as patients were sedated in the ICU, they were unable to communicate their visual symptoms [13, 19]. One study(4.55%) reported no changes in visual outcome [19]. Normal visual acuity was reported in one study [28].

### Posterior segment findings

The posterior segment of the eye, specifically the retina and choroid, was by far the most involved region (21; 95.45%) across the included studies [11–27, 29–32]. Chorioretinopathy was reported in 7(31.82%) studies [11–13, 15–17, 19], endophthalmitis was reported in 7(31.82%) studies [11, 13, 16, 19, 20, 23, 24], panophthalmitis was observed in one study(4.55%) [24] (Yaisawang et al.), reduced retinal vascular density was reported in one study(4.55%)(Simkiene et al.) [6], retinal detachment was reported in 3 studies(13.64%) [25, 29, 30]. Vitritis was reported in 3(13.64%) studies. [12, 14, 25] Other findings included vitreous cells [21], vitreous exudates [27], vitreous haemorrhage [30], and vitreous abscess [17]. The studies also reported rarely of choroiditis [14], panuveitis [24] and optic disc edema [18]. These manifestations were more commonly observed in fungal infections, but also seen in bacterial sepsis. However, 10 studies did not report retinal findings specific to sepsis.

### Anterior segment involvement

Anterior segment signs were described in 8(36.36%) studies [13, 16, 17, 19, 27, 31–33]. Indicators of ocular infection, like conjunctival congestion, were reported in 5(22.73%) studies [14, 21, 25, 27, 28], anterior chamber cells, flare, and hypopyon were reported in 6(27.27%) studies [14, 21, 25–27, 29]. Ramonas et al. [21, 24, 28, 30] reported one(4.55%) case of an iris abscess. These findings often coincided with posterior signs.

### Periocular and orbital findings

Periocular involvement was reported in 4(18.18%) studies. [21, 24, 28, 30] The findings included preseptal cellulitis [21, 24] proptosis [28], orbital cellulitis [24], and orbital haematoma [30]. In 3 (13.64%) of the studies, they were seen with posterior segment findings [21, 24, 30]. The above has been summarized in Table 2.

**Table 2:** Description of ocular findings in sepsis (*N* = 22)

Author	Visual morbidity	Anterior Segment	Posterior segment	Periocular
Nagasako et al. [11]	One patient (5.3%) with bacteremia had vision loss (no light perception); rest (94.7%) regained vision. There was no vision loss in the patient with candidemia.	-	34 patients (7.8%) developed retinal lesions (chorioretinitis or endophthalmitis).	-
Simkiene et al. [32]	NR	-	Significant decrease in retinal vascular length density in sepsis compared to normals (*p* < 0.001).	-
Huynh et al. [12]	One (2%) patient vision was 20/30 OD and 20/25 OS. After treatment, it was 20/25 OU, rest patients had normal vision	-	Chorioretinitis and vitritis.	-
Kirkegaard et al. [13]	Not reported as most patients were sedated in the ICU.	-	9 retinitis-infiltrative chorioretinal lesions without vitreous involvement, and one had endophthalmitis involving the vitreous body.	-
Santanaraman et al. [14]	Poor visual outcomes overall, with partial vision recovery in only two patients.	Cells and flare. Conjunctival congestion.	Vitreous haze, vitritis, and multifocal choroiditis	-
Mohammad et al. [19]	Two patients with Candida were either asymptomatic or unable to communicate symptoms. Visual acuity did not change significantly on follow-up visits after treatment.	-	Chorioretinitis or endophthalmitis identified in 4.3% (7 of 161)	-
Gluck et al. [15]	No reports of visual symptoms (38%)	-	One patient (2.9%), had chorioretinitis.	-
Yannis et al. [16]	Presenting visual acuity 20/20 to 20/800 in symptomatic cases. The final tested visual acuity ranged from 20 to 20/200.	-	Chorioretinitis was detected in 7 patients (5.6% rate), and endophthalmitis in 2 patients (1.6% rate).	-
Ilhami et al. [22]	Visual prognosis is not reported.	-	Retinal lesions were identified in 22 of the 150 patients (14.7%). The most common types of BRRLs included Roth’s spots, cotton-wool spots, and retinal hemorrhages.	-
Rodríguez-Adrián et al.[17]	Vision testing was not a primary outcome measure. Patients were asymptomatic at the time of retinal evaluation	-	Roth spots, retinal hemorrhages, and cotton wool spots. In candidemia, infiltrative chorioretinal lesions and vitreal abscesses	-
Chen et al. [23]	A better initial vision was associated with better final outcomes. Klebsiella pneumonia infection was associated with continued poor visual acuity.	-	Endogenous endophthalmitis	-
Yaisawang et al. [24]	Blindness in 11(73%) out of 15 cases. After treatment, blindness persisted in nine (64%) patients. Three patients (20%) had no light perception. Two (14%) had progressive vision loss. About 36% showed improved vision after treatment.	-	Endophthalmitis (four cases, 25%), panophthalmitis (two cases, 13%), and panuveitis (one case, 6%).	Orbital cellulitis (seven cases, 44%), preseptal cellulitis (two cases, 13%)
Ismail et al. [25]	Post-treatment, vision improved from counting fingers to 6/36	Right eye- conjunctival conjestion, anterior chamber inflammation, and hypopyon.	Vitritis and sub-retinal abscess on the nasal retina, about a four-disc diameters size, from 1 to 5 o’clock Inferior exudative retinal detachment	-
Amir et al. [18]	20/20 OD and NLP OS- remained same after systemic treatment	RAPD in left eye	Pallid disc edema with peripapillary hemorrhage in left eye	-
Krista et al. [26]	Both eyes 20/25 which decreased to 20/200 in the left eye. Final visual acuity 20/25 after treatment.	Hypopyon, and iris abscess with synechiae to the anterior lens capsule.	RE Fundus: Roth spots near the macula and in the periphery.	-
Saidi et al. [21]	Vision RE (OD) was counting finger, and left eye (OS) 6/60- > right eye CF to 6/60. LE vision of 6/18 unaided and 6/9 PH	Anterior chamber cells and flare, Conjunctival congestion.	Vitreous cells; Unifocal retinitis	Preseptal cellulitis
Suchit et al. [27]	Right eye normal, but Left eye: perceived light with an inaccurate direction of ray projection, later- no perception of light.	Conjunctival chemosis, corneal haze, and hypopyon	Yellow vitreous exudates Retina: no view Ultrasound: multiple medium-density echoes	-
Allegrini et al. [28]	Normal visual acuity at presentation.	Conjunctival chemosis, ophthalmoplegia	Normal	Proptosis, Imaging -dense and distended with thrombus in the superior ophthalmic vein with a non-fatty filling defect on the same side of the cavernous sinus.
Padmaja et al. [29]	PL+ both eyes- > post treatment, VA improved to 20/160 and 20/630	Corneal edema with Descemet layer folds Lens anterior capsule pigments, anterior chamber cells.	Both eyes: 360° of choroidal detachment with exudative retinal detachment touching the lens	-
Karinya et al. [30]	Light perception- after 2 years of treatment, final visual acuity was 6/36 OD and counting fingers OS.	NR	Bilateral dense vitreous hemorrhage, extensive sub-ILM hemorrhage, and tractional retinal detachments.	MRI scans demonstrated orbital hematomas.
Chang et al. [31]	At presentation her best-corrected visual acuity (BCVA) was 20/28 in her right eye and 20/133 in her left eye. After seven months of the operation, the BCVA in her left eye was 20/67	Conjunctival congestion Anterior chamber: cells and flare.	SD-OCT and fundoscopy revealed multiple intraretinal hemorrhages in the fovea, impending Macular Hole, and outer retinal disruption.	-
Penelope et al. [20]	First case, BE counting fingers progressed to light perception in both eyes. In the second case, the right eye was normal with pinhole acuity of 6/6 which changed to 6/36. Left visual acuity was hand movements, which reduced to light perception	NR	Both patients presented with severe endophthalmitis characterized by hemorrhagic necrotizing chorioretinitis, extensive retinal detachment, and infiltration of the choroid, retina, and vitreous with fungal hyphae	-

Table Footnote: BCVA: Best corrected visual acuity, ICU: Intensive Care Unit, NLP: No light perception, NR: Not reported, OD(RE): Right eye, OS(LE): Left eye OU(BE): Both eyes, Periocular findings: Preseptal cellulitis/Periorbital, PH: Pinhole, PL: Perception of Light, RAPD: Relative Afferent Pupillary Defect, SD-OCT: spectral-domain optical coherence tomography, VA: Visual acuity

### Change in management of sepsis with the presence of ocular manifestations

Indications for ocular examination were mentioned in 18(81.82%) studies [11–13, 15–21, 24–31]. The most common reason for the ophthalmology evaluation was positive blood culture in 8 (36.36%) studies [11–13, 15, 16, 19, 24, 25]. In 9 (40.91%) studies, the reason was the presence of visual complaints [11, 18–20, 25, 27, 29–31]. Ocular features being the presenting feature was reported in 5 (22.73%) studies [21, 25–28], routine eye evaluation was scheduled for sepsis patients in one study [17]. Infective endocarditis was the reason for suspecting ocular involvement in one study [11].

Management of sepsis with ocular manifestations was reported in 20(90.91%) studies [6, 11–16, 18–21, 23–31]. Among them, 13(59.08%) studies [12, 14, 16, 20, 21, 23–27, 29–31] reported ocular management along with systemic treatment. Topical antibiotics were reported in 2 (9.09%) studies [26, 27], intravitreal antifungals were administered in three(13.64%) studies [12, 16, 20], intravitreal antibiotics were administered in 6(27.27%) studies [14, 20, 21, 23, 25, 26], and vitrectomy was performed in 8(36.36%) studies [14, 16, 23–25, 29–31]. Other surgeries performed included choroidal drainage, drainage retinotomy (Padmaja et al.) [29], grid laser surgery for macular edema (Saidi et al.) [21], and retinal detachment surgery for proliferative vitreoretinopathy (Karinya et al.). [30] When the eye condition worsened, enucleation was done in one study [24] and evisceration was done in another [27].

Systemic management included intravenous antifungal treatment in 6(27.27%) studies [12, 13, 15, 16, 19, 20], intravenous antibiotic treatment in 10(45.45%) studies. [11, 14, 21, 25–29, 31] Other management of shock included mechanical ventilation, noradrenaline (Simkiene et al.) [6], stopping anti-hypertensive agents, salt tablets, midodrine and oral rehydration (Amir et al.). [18] Source of infection, such as infected teeth, was removed in one study after treatment of the infection (Allegrini et al.) [28].

Ten (45.45%) studies reported systemic and ocular treatment [6, 12, 14, 16, 20, 21, 25, 27, 29, 31]. Few studies reported that when ocular findings were present, they had extended the duration [15] or increased the dosage of systemic antifungals. [16] Further details of the systemic and ocular treatment have been described in Supplementary Table 4.

## Summary

Ocular involvement was present in all studies. Bacteriological origin (14; 63.64% of studies) was reported as the most common organism of infection, followed by fungus (10; 45.45% of studies). The urinary tract was the most common source of sepsis (7; 31.82% studies), followed by central venous catheter (5; 22.73% of studies), leading to ocular manifestations. Indications for ocular examination were mentioned in 18 (81.82%) studies, with positive blood culture in 8 (36.36%) studies being reported as the reason for ocular examination. Visual impairment was reported in 15 (68.18%) studies. In 2 studies (9.09%), as patients were sedated, they could not communicate their ocular symptoms till late. Regarding ocular manifestations, the posterior segment was commonly involved (21 studies, 95.45%), with chorioretinitis and endophthalmitis being present (each in 7; 31.82% studies), followed by the anterior segment (8 studies; 36.36%) and periocular (4 studies, 18.18%) involvement. Management of sepsis with ocular manifestations was reported in 20(90.91%) studies. Ocular treatment involved topical antibiotics (9.09%), intravitreal antibiotics (6; 27.27%) and intravitreal antifungals (3; 13.64%). Vitrectomy was performed in 8(36.36%) studies. Systemic antibiotics (10 studies; 45.45%), systemic antifungals (6 studies; 27.27%), supported ventilation, hemodynamic management (1), and removal of the source of infection (1) were also reported.

## Discussion

In sepsis, infection can manifest in the eye in several ways. Early detection by an ophthalmologist may alter both visual and systemic outcomes, especially in ICU settings where there are challenges in the evaluation of critically ill patients. The narrative review by Khan et al. [1] on endogenous endophthalmitis (2007) and a case-based synthesis by Schiedler et al. [33], primarily emphasized that bloodstream infections, especially fungal etiologies, can cause endophthalmitis and blindness. As sepsis can be caused by varied etiologies and management guidelines have evolved over the years, this scoping review highlights the various presentations of ocular infections in sepsis. Secondly, we also described the changes in sepsis management in the presence of ocular findings.

Previous reviews on septic microangiopathy have highlighted Candida species [34, 35] as frequent causative agents of endogenous fungal endophthalmitis in immunocompromised patients. Bacterial sepsis, transmitted through the hematogenous route, has also been associated with retinopathy through the release of toxins. In our review, we found a mixture of fungal and bacterial pathogens across studies, confirming that though fungal infections remain of concern, bacterial sepsis also contributes to ocular morbidity. Staphylococcus infections were the second most common etiology after Candida, and gram-negative bacteria also featured prominently.

Our review reports that gram-positive organisms are frequently implicated in developed nations, whereas gram-negative bacteria, especially *Klebsiella pneumoniae* and *Pseudomonas aeruginosa*, are commonly associated with ocular seeding in lower- and middle-income countries (LMICs). [36] These variations may reflect differences in ICU protocols, regional microbiological profiles, and patterns of antimicrobial resistance.

Our review provides a structured ophthalmic categorization of ocular findings among sepsis studies. Anterior segment signs like hypopyon (6; 27.27%) [14, 21, 25–27, 29] and conjunctival congestion (5; 22.73%) [14, 21, 25, 27, 28], were less commonly described in studies, likely due to limited examination in sedated patients or reporting bias [12, 33]. The posterior segment was the most affected area(21; 95.45%) across the included studies [11–27, 29–32] in sepsis. The most frequent finding was chorioretinitis, reported in 7(31.82%) studies [11–13, 15–17, 19] and endophthalmitis, which was reported in 7(31.82%) studies [11, 13, 16, 19, 20, 23, 24]. Early ophthalmological evaluation is important in fungemia, noting that 57% patients with chorioretinitis or endophthalmitis reported no visual symptoms [16]. In our review, the common occurrence of posterior segment lesions in fungal and bacterial sepsis highlighted the critical need for routine fundus screening in high-risk sepsis cohorts.

Rodríguez-Adrián et al. reported that some retinal signs, such as cotton-wool spots, can be nonspecific and often overlap with conditions like diabetes or hypertension. The diagnostic yield improved when lesions appeared newly, and indicated systemic decline [17], worsening sepsis or hospital-acquired infections. [22] Preseptal cellulitis or orbital inflammation can also be an early indicator of systemic infection [27]; but they were largely underexplored in the studies included. In rare cases, eye involvement preceded general signs of sepsis [21, 25, 26], suggesting that fundus examination could serve as a potential early warning tool. Hence, timely eye consultations in ICU patients with sepsis, are vital to prevent ocular complications.

Our review reports that the urinary tract was reported as the most common source of sepsis (7; 31.82% studies), followed by the central venous catheter (5 (22.73%) studies), leading to ocular manifestations. The involvement of the soft tissue, gastrointestinal, infective endocarditis, nasal wall, and dental abscesses represents a diverse systemic focus that can lead to ocular complications. Previous reviews support our report, stating that systemic bacteriuria can seed the vascular choroid and retinal vasculature, leading to blinding conditions like endogenous endophthalmitis [37–39]. This highlights the importance of collaboration between ophthalmologists and specialists, including internal medicine experts and internists, for comprehensive management.

Management of sepsis with ocular manifestations was reported in 20(90.91%) studies. Ocular treatment included topical antibiotics (9.09%), intravitreal antibiotics (27.27%) and antifungals (3 studies; 13.64%) and vitrectomy (8 studies; 36.36%). Detecting posterior segment involvement early, especially in patients with fungemia, allowed for targeted removal of the focus of infection in those refractory to medical treatment. Along with systemic antibiotics (10 studies; 45.45%) and antifungals (6 studies; 27.27%), ventilation support, hemodynamic management, source control and supportive care, in accordance with sepsis guidelines and reviews, this facilitates improvements in organ function, reduction of systemic inflammation, and enhanced survival rates [40–42].

The review has limitations. Among the 22 studies included, due to the rare occurrence of the pathologies studied, most were case reports or small case series [18, 20–22, 26–30] and only three studies [13, 16, 32] employed prospective observational or cohort designs. The evidence base is limited, with the predominant inclusion of case reports and small retrospective series, which introduces a high risk of reporting and publication bias, thereby limiting the ability to make strong, generalizable conclusions about incidence or prevalence. The scoping review design does not include a quality assessment (risk of bias) or meta-analysis, which limits the strength of the quantitative conclusions. The exclusion of non-English articles also potentially omits relevant data from regions with high sepsis burdens. There was heterogeneity in the definitions of Sepsis 2 or Sepsis 3 followed among studies across the years, potentially affecting the analysis of studies involving different patient cohorts. Standardized ocular examinations or imaging protocols were seldom followed, and very few reported anterior segment findings or long-term follow-up data. Variations in reporting, small sample sizes, and the limited use of quantitative evaluation tools lead to underreporting of eye symptoms in sepsis, which remain significant limitations that future research should address.

The included studies provided valuable insight into pathogen distribution, clinical signs, and management protocols with sepsis. Studies from various geographic areas allowed for a global comparison of microbiological patterns. Differences in microbiological causes based on region highlight the need for careful diagnostic attention. The use of non-invasive imaging techniques like fundus photography or optical coherence tomography (OCT) in some reports improved diagnostic clarity by reflecting systemic microcirculatory compromise. We recommend incorporating routine bedside fundus exams into sepsis evaluation protocols, especially in ICU settings. All patients with fungemia or unexplained fever and visual changes should receive prompt eye assessments. Collaborative care involving eye specialists and critical care doctors may improve early detection and enhance both visual and systemic outcomes. Future research should focus on prospective, multicenter studies with standardized eye evaluations in sepsis.

## Conclusion

From this review, we conclude that ocular manifestations constitute a serious complication in sepsis, characterized by frequent reports of visual impairment, involvement of the posterior segment and mixed etiology consisting of bacterial followed by fungal etiology with common sources of infection being the urinary tract. Early detection in at-risk sepsis patients with positive blood cultures, combining ocular and systemic interventions, can aid in improving outcomes.

## Electronic supplementary material

Below is the link to the electronic supplementary material.

Supplementary material 1

## Data Availability

All the data and materials related to the study is presented in the Supplementary Files and main table.

## Abbreviations

BRRLs: Bacteremia-related retinal lesions
BCVA: Best corrected visual acuity
CRAE: Central retinal arteriolar equivalent
DBCI: Disseminated bacterial or candidal infections
DIC: Disseminated Intravascular Coagulation
EE: Endogenous endophthalmitis
ICU: Intensive Care Unit
ILM: Internal Limiting Membrane
LMICs: Lower- and middle-income countries
MH: Macular hole
MSSA: Methicillin-Sensitive Staphylococcus Aureus
MRI: Magnetic Resonance Imaging
MeSH: Medical Subject Headings
NA: Not assessed
NLP: No light perception
NR: Not reported
OCT: Optical Coherence Tomography
OD(RE): Right eye
OS(LE): Left eye
OU: Both eyes
PL: Perception of Light
PPV: Pars plana vitrectomy
PRESS: Peer Review of Electronic Search Strategies
PVR: Proliferative vitreoretinopathy
PH: Pinhole
PRISMA: Preferred Reporting Items for Systematic Reviews and Meta-Analyses
SD-OCT: Spectral-domain optical coherence tomography
SOFA: Sequential Organ Failure Assessment
USA: United States of America
UTI: Urinary Tract Infection

## References

[1] Khan A, Okhravi N, Lightman S. The eye in systemic sepsis. Clin Med. 2002;2:444–48. 10.7861/clinmedicine.2-5-444 PMC4953086 12448593

[2] Singer M, Deutschman CS, Seymour CW, et al. The third International consensus definitions for sepsis and septic shock (sepsis-3). JAMA. 2016;315:801–10. 26903338 10.1001/jama.2016.0287 PMC4968574

[3] Pirani V, Pelliccioni P, De Turris S, et al. The eye as a window to systemic infectious diseases: old enemies, new imaging. J Clin Med. 2019;8:1392. 31492008 10.3390/jcm8091392 PMC6780210

[4] Bourne RRA, Adelson J, Flaxman S, et al. Global prevalence of blindness and distance and near vision impairment in 2020: progress towards the vision 2020 targets and what the future holds. Invest Ophthalmol Vis Sci. 2020;61:2317–2317.

[5] Breazzano MP, Day HR Jr, Bloch KC, et al. Utility of ophthalmologic screening for patients with Candida bloodstream infections: a Systematic review. JAMA Ophthalmol. 2019;137:698–710. 30998819 10.1001/jamaophthalmol.2019.0733

[6] Simkiene J, Pranskuniene Z, Vitkauskiene A, et al. Ocular microvascular changes in patients with sepsis: a prospective observational study. Ann Intensive Care. 2020;10:38. 32266602 10.1186/s13613-020-00655-x PMC7138894

[7] Tricco AC, Lillie E, Zarin W, et al. PRISMA Extension for scoping reviews (PRISMA-ScR): checklist and explanation. Ann Intern Med. 2018;169:467–73. 30178033 10.7326/M18-0850

[8] Arksey H, O’Malley L. Scoping studies: towards a methodological framework. Int J Soc Res Methodol. 2005;8:19–32.

[9] Clark JM, Sanders S, Carter M, et al. Improving the translation of search strategies using the polyglot search Translator: a randomized controlled trial. J Med Libr Assoc. 2020;108:195–207. 32256231 10.5195/jmla.2020.834 PMC7069833

[10] McGowan J, Sampson M, Salzwedel DM, et al. PRESS Peer review of electronic search Strategies: 2015 guideline statement. J Clin Epidemiol. 2016;75:40–46. 27005575 10.1016/j.jclinepi.2016.01.021

[11] Nagasako Y, Inagaki K, Serizawa S, et al. Risk factors associated with retinal lesions resulting from widespread systemic infection. Ophthalmol Retina. 2017;1:333–38. 31047520 10.1016/j.oret.2016.12.011

[12] Nancy H, Hy C, Sheila B-G. Ocular involvement in hospitalized patients with candidemia: analysis at a Boston tertiary care center. Ocul Immunol Inflammation. 20. 10.3109/09273948.2011.646383. Epub ahead of print April 2012. 10.3109/09273948.2011.646383 22409562

[13] Kirkegaard Karmisholt M, Hjort U, Loumann Knudsen L, et al. Candidaemia and risk of intraocular infection: a Danish hospital-based cohort study. Scand J Infect Dis. 2008;40:241–46. 17852897 10.1080/00365540701642120

[14] Santanaraman R, Ramalingam R, Rangarajan D, et al. Endogenous endophthalmitis in the setting of kidney disease: a case series - PMC. https://pmc.ncbi.nlm.nih.gov/articles/PMC11619030/. (accessed 28 July 2025. 10.25259/IJN_271_2024 PMC11619030 39649322

[15] Gluck S, Headdon WG, Tang D, et al. The Incidence of ocular candidiasis and evaluation of routine ophthalmic examination in critically ill patients with Candidaemia. 10.1177/0310057X1504300605. 10.1177/0310057X1504300605 26603792

[16] Yannis P, C S, Pa K, et al. Prospective trial of endogenous fungal endophthalmitis and chorioretinitis rates, clinical course, and outcomes in patients with fungemia. Retina (Philadelphia, Pa). 36. 10.1097/IAE.0000000000000919. Epub ahead of print July 2016. 10.1097/IAE.0000000000000919 26655621

[17] Rodríguez-Adrián LJ, Rt K, Lg T-D, et al. Retinal lesions as clues to disseminated bacterial and candidal infections: frequency, natural history, and etiology. Medicine. 82. 10.1097/01.md.0000076008.64510.f1. Epub ahead of print May 2003. 10.1097/01.md.0000076008.64510.f1 12792305

[18] Vosoughi AR, Micieli JA. Preservation of vision after early recognition of anterior ischemic optic neuropathy in a patient with sepsis. Case Rep Ophthalmol. 2023;14:314–18. 37485244 10.1159/000530326 PMC10359668

[19] Mohammad S, Gm G, Kt A, et al. Incidence of chorioretinitis and endophthalmitis in hospitalized patients with fungemia. Eye (Lond, Engl). 36. 10.1038/s41433-021-01477-2. Epub ahead of print January 2022. 10.1038/s41433-021-01477-2 PMC8727606 33686234

[20] McKelvie PA, Wong EY, Chow LP, et al. Scedosporium endophthalmitis: two fatal disseminated cases of Scedosporium infection presenting with endophthalmitis. Clin Exp Ophthalmol. 2001;29:330–34. 11720162 10.1046/j.1442-9071.2001.00444.x

[21] Saidi NA, Ngoo QZ, Jusoh S, et al. Endogenous endopthalmitis in disseminated Methicillin-Sensitive Staphylococcus aureus (MSSA) bacteremia. Cureus. 15:e34707. 10.7759/cureus.34707 PMC9995743 36909129

[22] Ilhami C, Cihangiroglu M, Yilmaz T, et al. The prevalence of bacteraemia-related retinal lesions in seriously ill patients. J Infect. 52. 10.1016/j.jinf.2005.04.006. Epub ahead of print February 2006. 10.1016/j.jinf.2005.04.006 15904970

[23] Chen Y-J, Kuo H-K, Wu P-C, et al. A 10-year comparison of endogenous endophthalmitis outcomes: an east Asian experience with Klebsiella pneumoniae infection. RETINA. 2004;24:383–90. 15187660 10.1097/00006982-200406000-00008

[24] Yaisawang S, S A, P C, et al. Ocular involvement in melioidosis: a 23-year retrospective review. J Ophthalmic Inflammation Infect. 2018, March, 27;8. Epub ahead of print 10.1186/s12348-018-0147-6. 10.1186/s12348-018-0147-6 PMC5869345 29589220

[25] Mohd-Ilham I, Zulkifli M, Yaakub M, et al. A case of a large sub-retinal abscess secondary to Klebsiella pneumoniae endophthalmitis in a pyelonephritis patient. Cureus. 2019;11:e 4656. 10.7759/cureus.4656 PMC6625674 31316877

[26] Krista R, Benjamin D F. Iris abscess as an unusual presentation of endogenous endophthalmitis in a patient with bacterial endocarditis. Am J Ophthalmol. 135. 10.1016/s0002-9394(02)01935-9. Epub ahead of print February 2003. 10.1016/s0002-9394(02)01935-9 12566031

[27] Dadia SD, Modi RR, Shirwadkar S, et al. Salmonella typhi associated endogenous endophthalmitis: a case report and a review of literature. Ocul Immunol Inflammation. 2018;26:527–32. 10.1080/09273948.2017.1306085 28453408

[28] Allegrini D, Reposi S, Nocerino E, et al. Odontogenic orbital cellulitis associated with cavernous sinus thrombosis and pulmonary embolism: a case report. J Med Case Rep. 11. 10.1186/s13256-017-1309-0. Epub ahead of print 20 June 2017. 10.1186/s13256-017-1309-0 PMC5477346 28629401

[29] Padmaja R, A V, S S, et al. Bilateral choroidal detachment with exudative retinal detachment in a patient with septicaemia. BMJ Case Rep. 2016. 10.1136/bcr-2016-216886. Epub ahead of print 20 December 2016. 10.1136/bcr-2016-216886 PMC5174762 27999127

[30] Karinya L, En H, Th W. Severe ocular involvement in disseminated intravascular coagulation complicating meningococcaemia. Graefe’s Archiv Clin Exp Ophthalmol = Albrecht von Graefes Arch fur Klin und experimentelle Ophthalmologie. 243. 10.1007/s00417-005-1155-4. Epub ahead of print October 2005. 10.1007/s00417-005-1155-4 15838663

[31] Chang HY, Kyu Hyung P, Se Joon W. Macular hole formation secondary to bacterial septic embolism demonstrated by serial spectral-domain optical coherence tomography imaging. Ocul Immunol Inflammation. 21. 10.3109/09273948.2012.752014. Epub ahead of print April 2013. 10.3109/09273948.2012.752014 23697861

[32] Jurate S, Zivile P, Martynas P, et al. Alterations of retinal vessels in patients with sepsis. J Clin Monit Comput. 34. 10.1007/s10877-019-00401-0. Epub ahead of print October 2020. 10.1007/s10877-019-00401-0 31650429

[33] Schiedler V, Scott IU, Flynn HW, et al. Culture-proven endogenous endophthalmitis: clinical features and visual acuity outcomes. Am J Ophthalmol. 2004;137:725–31. 15059712 10.1016/j.ajo.2003.11.013

[34] Callegan MC, Engelbert M, Parke DW, et al. Bacterial endophthalmitis: epidemiology, therapeutics, and bacterium-host interactions. Clin Microbiol Rev. 2002;15:111–24. 11781270 10.1128/CMR.15.1.111-124.2002 PMC118063

[35] Gentile P, Ragusa E, Bruno R, et al. Endogenous Candida Endophthalmitis: an update on epidemiological, Pathogenetic, clinical, and therapeutic aspects. Ocul Immunol Inflamm. 2025;1–17. 10.1080/09273948.2025.2524013 40638241

[36] Tiecco G, Laurenda D, Mulè A, et al. Gram-negative endogenous endophthalmitis: a Systematic review. Microorganisms. 2022;11:80. 36677371 10.3390/microorganisms11010080 PMC9860988

[37] Khairallah M, Abroug N, Smit D, et al. Systemic and ocular manifestations of arboviral infections: a review. Ocul Immunol Inflammation. 2024;32:2190–208. 10.1080/09273948.2024.2320724 38441549

[38] Mabra D, Yeh S, Shantha JG. Ocular manifestations of bartonellosis. Curr Opin Ophthalmol. 2018;29:582–87. 30124532 10.1097/ICU.0000000000000522

[39] Andrzej G, B P, Sj K. Microbial flora and resistance in ophthalmology: a review. Graefe’s Archiv Clin Exp Ophthalmol = Albrecht von Graefes Arch fur Klin und experimentelle Ophthalmologie. 255. 10.1007/s00417-017-3608-y. Epub ahead of print May 2017. 10.1007/s00417-017-3608-y PMC5394129 28229218

[40] Dellinger RP, Carlet JM, Masur H, et al. Surviving sepsis campaign guidelines for management of severe sepsis and septic shock. Crit Care Med. 2004;32:858–73. 15090974 10.1097/01.ccm.0000117317.18092.e4

[41] Thompson K, Venkatesh B, Finfer S. Sepsis and septic shock: current approaches to management. Intern Med J. 2019;49:160–70. 30754087 10.1111/imj.14199

[42] Sessler CN, Perry JC, Varney KL. Management of severe sepsis and septic shock. Curr Opin Crit Care. 2004;10:354–63. 15385751 10.1097/01.ccx.0000139363.76068.7b

