# Supplementary material for: Ocular features in sepsis - a scoping review
Source: BMC Ophthalmol. 2026 Feb 7;26:120. doi: 10.1186/s12886-026-04647-6 (PMC12977651; doi:10.1186/s12886-026-04647-6)
Supplement: Supplementary file 1 — Supplementary material 1 [file 12886_2026_4647_MOESM1_ESM.docx]

**Ocular Features in Sepsis - A Scoping Review**

**Supplementary Files**

**Supplementary Table 1: Preferred Reporting for Systematic reviews and Meta-Analyses extension for Scoping Reviews (PRISMA-ScR) Checklist.**

| **SECTION** | **ITEM** | **PRISMA-ScR CHECKLIST ITEM** | **REPORTED ON PAGE #** |
| --- | --- | --- | --- |
| **TITLE** | | | |
| Title | 1 | Identify the report as a scoping review. | 1 |
| **ABSTRACT** | | | |
| Structured summary | 2 | Provide a structured summary that includes (as applicable): background, objectives, eligibility criteria, sources of evidence, charting methods, results, and conclusions that relate to the review questions and objectives. | 2 |
| **INTRODUCTION** | | | |
| Rationale | 3 | Describe the rationale for the review in the context of what is already known. Explain why the review questions/objectives lend themselves to a scoping review approach. | 4 |
| Objectives | 4 | Provide an explicit statement of the questions and objectives being addressed with reference to their key elements (e.g., population or participants, concepts, and context) or other relevant key elements used to conceptualize the review questions and/or objectives. | 4-5 |
| **METHODS** | | | |
| Protocol and registration | 5 | Indicate whether a review protocol exists; state if and where it can be accessed (e.g., a Web address); and if available, provide registration information, including the registration number. | 5 |
| Eligibility criteria | 6 | Specify characteristics of the sources of evidence used as eligibility criteria (e.g., years considered, language, and publication status), and provide a rationale. | 6 |
| Information sources* | 7 | Describe all information sources in the search (e.g., databases with dates of coverage and contact with authors to identify additional sources), as well as the date the most recent search was executed. | 7 |
| Search | 8 | Present the full electronic search strategy for at least 1 database, including any limits used, such that it could be repeated. | Supplementary File: Table 1 |
| Selection of sources of evidence | 9 | State the process for selecting sources of evidence (i.e., screening and eligibility) included in the scoping review. | 7 |
| Data charting process | 10 | Describe the methods of charting data from the included sources of evidence (e.g., calibrated forms or forms that have been tested by the team before their use, and whether data charting was done independently or in duplicate) and any processes for obtaining and confirming data from investigators. | 8 |
| Data items | 11 | List and define all variables for which data were sought and any assumptions and simplifications made. | 5-6 |
| Critical appraisal of individual sources of evidence | 12 | If done, provide a rationale for conducting a critical appraisal of included sources of evidence; describe the methods used and how this information was used in any data synthesis (if appropriate). | Not done as non-mandatory |
| Synthesis of results | 13 | Describe the methods of handling and summarizing the data that were charted. | 8 |
| **RESULTS** | | | |
| Selection of sources of evidence | 14 | Give numbers of sources of evidence screened, assessed for eligibility, and included in the review, with reasons for exclusions at each stage, ideally using a flow diagram. | 9 |
| Characteristics of sources of evidence | 15 | For each source of evidence, present characteristics for which data were charted and provide the citations. | 10 |
| Critical appraisal within sources of evidence | 16 | If done, present data on critical appraisal of included sources of evidence (see item 12). | Not applicable |
| Results of individual sources of evidence | 17 | For each included source of evidence, present the relevant data that were charted that relate to the review questions and objectives. | 8-11 |
| Synthesis of results | 18 | Summarize and/or present the charting results as they relate to the review questions and objectives. | 10-14 |
| **DISCUSSION** | | | |
| Summary of evidence | 19 | Summarize the main results (including an overview of concepts, themes, and types of evidence available), link to the review questions and objectives, and consider the relevance to key groups. | 14 |
| Limitations | 20 | Discuss the limitations of the scoping review process. | 17 |
| Conclusions | 21 | Provide a general interpretation of the results with respect to the review questions and objectives, as well as potential implications and/or next steps. | 18 |
| Funding | 22 | Describe sources of funding for the included sources of evidence, as well as sources of funding for the scoping review. Describe the role of the funders of the scoping review. | 20 |

JBI = Joanna Briggs Institute; PRISMA-ScR = Preferred Reporting Items for Systematic reviews and Meta-Analyses

**Supplementary Table 2**: Search Strategy utilized for electronic databases (N= 2276)

| PubMed |  | Records Obtained | Date of Search |
| --- | --- | --- | --- |
| Sepsis(1) | ("Sepsis"[Mesh] OR Septicemi*[Text] OR Septicaemi*[Text] OR “Septic Shock”[text] OR "Shock, Septic"[Mesh]) | 181,276 | 29.12.25 |
| Eye(2) | ("Eye"[Mesh] OR “Eye disease*"[text] OR Ocular[text] OR "Ocular disease*"[text] OR "Vision disorder*"[text] OR "Visual impairment"[text] OR "Vision,low"[text] OR "Refractive error*"[text] OR “Ophthalm*”[text] OR Conjunctiv*[text] OR Conjunctiva[Mesh] OR Cornea "Corneal Ulcer"[Mesh] OR “Corneal dis*”[text] OR “Cataract”[Mesh] OR "Uveitis"[Mesh] OR "Retinal Diseases"[Mesh] OR "Optic Nerve Diseases"[Mesh] OR "Endophthalmitis"[Mesh]) | 286,386 | 29.12.25 |
| Combination of 1 and 2 | **(("Sepsis"[Mesh] OR Septicemi*[Text] OR Septicaemi*[Text] OR "Septic Shock"[text] OR "Shock, Septic"[Mesh])) AND (("Eye"[Mesh] OR "Eye disease*"[text] OR Ocular[text] OR "Ocular disease*"[text] OR "Vision disorder*"[text] OR "Visual impairment"[text] OR "Vision,low"[text] OR "Refractive error*"[text] OR "Ophthalm*"[text] OR Conjunctiv*[text] OR Conjunctiva[Mesh] OR "Corneal Ulcer"[Mesh] OR "Corneal dis*"[text] OR "Cataract"[Mesh] OR "Uveitis"[Mesh] OR "Retinal Diseases"[Mesh] OR "Optic Nerve Diseases"[Mesh] OR "Endophthalmitis"[Mesh]))** Filters: **English, Adults aged 18 years above, year 2000 till 29.12.2025** | 408 | 29.12.25 |
| Scopus | TITLE-ABS-KEY ( ( ( Sepsis OR Septicemi* OR Septicaemi* OR "Septic Shock" OR "Shock, Septic" ) ) AND ( ( "Eye" OR "Eye disease*" OR Ocular OR "Ocular disease*" OR "Vision disorder*" OR "Visual impairment" OR "Vision,low" OR "Refractive error*" OR "Ophthalm*" OR Conjunctiv* OR Conjunctiva OR Cornea "Corneal Ulcer" OR "Corneal dis*" OR "Cataract" OR "Uveitis" OR "Retinal Diseases" OR "Optic Nerve Diseases" OR "Endophthalmitis" ) ) ) AND ( LIMIT-TO ( LANGUAGE , "English" ) )Adults and Humans | 583 | 29.12.25 |
| Embase | ('sepsis':ti,ab OR 'septicemi*':ti,ab OR 'septicaemi*':ti,ab OR 'septic shock':ti,ab) AND ('eye':ti,ab OR 'eye disease*':ti,ab OR 'ocular':ti,ab OR 'ocular disease*':ti,ab OR 'vision disorder*':ti,ab OR 'visual impairment':ti,ab OR 'vision,low':ti,ab OR 'refractive error*':ti,ab OR 'ophthalm*':ti,ab OR 'conjunctiv*':ti,ab OR 'cornea ulcer':ti,ab OR 'corneal dis*':ti,ab OR 'cataract':ti,ab OR 'uveitis':ti,ab OR 'retina disease':ti,ab OR 'optic nerve disease':ti,ab OR 'endophthalmitis':ti,ab) AND [english]/**lim : English, Adults aged 18 years above, year 2000 till 29.12.2025** | 935 | 29.12.25 |
| ProQuest | Nursing and Allied Health Database, Public Health Database  Health and Medical collection, Coronavirus database  abstract((Sepsis OR Septicaemi* OR "Septic Shock") AND (Eye OR "Eye disease*" OR Ocular OR "Ocular disease*" OR "Visual impairment" OR "Refractive error*" OR "Ophthalm*" OR Conjunctiv* OR "Corneal Ulcer" OR "Corneal dis*" OR "Cataract" OR "Uveitis" OR "Retinal Diseases" OR "Optic Nerve Diseases" OR "Endophthalmitis")) English Filter | 350 | 29.12.25 |
|  | **Total** | 2276 |  |

**Supplementary Table 3**: General study characteristics of included studies (N=22)

| **Author, Journal, Study Year, Country** | **Sample Size** | **Study design and Objective** |
| --- | --- | --- |
| Nagasako et al.  Ophthalmol Retina  2017, Japan^11^ | 433 inpatients with positive blood culture results | Retrospective case series to investigate the frequency and risk factors associated with retinal lesions, including chorioretinitis and endophthalmitis, in patients with systemic infections. |
| Simkiene,^32^  Lithuania  J Clin Monit Comput. 2020 | 60 participants: 40 patients with sepsis or septic shock and 20 age-matched healthy controls | Single-center prospective observational study to compare the retinal vasculature of septic patients with age-matched healthy volunteers. |
| Huynh et al.^12^  United States  Ocul Immunol Inflam.2012 | 118 hospitalized patients were diagnosed with candidemia. | Retrospective observational study to determine the prevalence of ocular involvement in hospitalized patients with candidemia and to assess the need for routine ophthalmologic consultations for these patients. |
| Kirkegaard et al.^13^  Denmark Scand J Infect Dis.2008 | 203 cases of candidemia, with 86 patients undergoing ophthalmologic examination. | Cohort study conducted over a 10-year period (1995–2004 to assess the current risk and prognosis of intraocular infection in Danish candidemia patients and to determine the frequency of intraocular infection, associated ophthalmologic findings, and the outcome. |
| Santanaraman et al. ^14^India, Indian J Nephrol. 2024 | Five patients with kidney disease who developed EE between 2015 and 2023. | Case series to discuss the clinical profile, management, and outcomes of five patients with kidney disease who developed endogenous endophthalmitis (EE) in the context of sepsis. |
| Mohammad, ^19^USA, Eye (Lond).2022 | 161 patients with positive fungal cultures | Retrospective chart review to determine the incidence of chorioretinitis and endophthalmitis among hospitalized patients with fungemia, and to evaluate the effectiveness of routine dilated fundus examinations. |
| Gluck et al. ^15^Australia, Anaesth Intensive Care  . 2015 | 93 patients with candidemia were included based on blood culture results. | Ten-year retrospective observational study to estimate the incidence of ocular candidiasis in critically ill patients with candidemia and evaluate the impact of routine ophthalmic examinations on patient management. |
| Yannis et al.^16^,  USA, Retina. 2016 | 125 patients enrolled, with fungemia confirmed by positive blood cultures. | Prospective cohort study to determine the rate and risk factors for endogenous chorioretinitis and endophthalmitis in patients with fungemia. |
| Ilhami et al.^22^  Turkey, J Infect  . 2006 | 150 adult hospitalized critically ill patients. | Observational study to investigate the frequency, etiology, and risk factors of retinal lesions in patients with bacteremia and sepsis. |
| Rodríguez-Adrián et al.^17^, USA, Medicine. 2003 | Weekly funduscopic examinations of 77 ICU patients | Observational study to understand the frequency, natural history, and etiology of retinal lesions in patients with disseminated bacterial or candidal infections (DBCI) and assess the diagnostic utility of ophthalmologic consultation in critical care. |
| Chen et al. ^23^Taiwan, Retina. 2004 | 74 patients with 86 eyes treated for endogenous endophthalmitis from July 1992 to June 2002. | Retrospective review to evaluate the sources of infection and causative organisms in endogenous endophthalmitis cases, focusing on outcomes for patients with Klebsiella pneumoniae infection over a 10-year period. |
| Yaisawang et al.^24^, Thailand, J Ophthalmic Inflamm Infect. 2018 | 16 cases of ocular involvement from a cohort of melioidosis patients from 1993 to 2016. | Retrospective review to estimate the prevalence and investigate the clinical presentations, management, and visual outcomes of ocular involvement in patients with melioidosis in Khon Kaen, Thailand. |
| Ismail et al.^25^, Malaysia, Cureus. 2019 | One patient, a 39-year-old female. | Case report of endophthalmitis in a pyelonephritis patient |
| Amir et al.^18^, Canada  Case Rep Ophthalmol 2023 | One patient, a 61-year-old male. | To report a case of both eyes NAION and visual loss in the left eye secondary to hypotension in septic shock |
| Krista et al.^26^ USA Am J Ophthalmol. 2003 | One patient, a 37-year-old male intravenous drug user. | To report a case of endogenous endophthalmitis, a patient with bacterial endocarditis with a septic metastasis to the iris. |
| Saidi et al. ^21^Malaysia Cureus. 2023 | One patient (43-year-old male) | To report a case of poorly controlled diabetes mellitus who was admitted for bilateral nasoseptal cellulitis with a right nasal wall abscess and right vocal cord palsy who developed endophthalmitis |
| Suchit et al^27^ India Ocul Immunol Inflamm. 2018 | One patient, a 28-year-old immunocompetent male. | To report a rare case of Salmonella typhi associated endogenous endophthalmitis in an immunocompetent male |
| Allegrini et al.^28^ Italy, J Med Case Rep. 2017 | One patient, a 46-year-old woman. | To report a case of orbital cellulitis with systemic infectious etiology |
| Padmaja et al.^29^ India. BMJ Case Rep. 2016 | One patient, a 39-year-old man | To describe a case of bilateral choroidal and exudative retinal detachment with hypotony in a patient with septicaemia. |
| Karinya et al. ^30^United Kingdom, Graefes Arch Clin Exp Ophthalmol  . 2005 | One patient, an 18-year-old female | To report a case of a female with meningococcal septicaemia and DIC who developed bilateral dense vitreous haemorrhage. |
| Chang et al.^31^ South Korea, Ocul Immunol Inflamm  . 2013 | One patient, a 69-year-old woman. | To describe a case of macular hole (MH) formation secondary to bacterial septic embolism and demonstrate the changes thereof using serial spectral-domain optical coherence tomography (SD-OCT) images |
| Penelope et al. Australia,^20^ Clinical & Experimental Ophthalmology, 2001 | Two patients | To report cases of acute myeloid leukemia with disseminated infection with Scedosporium who presented with endophthalmitis |

Table Footnote: Totally 1526 patients were included among 22 studies.

DBCI: disseminated bacterial or candidal infections DIC: Disseminated Intravascular Coagulation, EE: endogenous endophthalmitis, ICU: Intensive Care Unit, MH: macular hole, NAION: Non-arteritic Anterior Ischemic Optic Neuropathy, SDOCT: spectral-domain optical coherence tomography

**Supplementary Table 4**: Change in intervention based on the presence of ocular findings and results reported in the study(N=22)

| **Author** | **Indication for Ocular examination** | **Management** | **Results** |
| --- | --- | --- | --- |
| Nagasako et al.^11^ | - Infective endocarditis - Blood culture positive - Visual complaints | Dialysis: 6 (17.7) cases had retinal lesions  Steroid use: 16 (47.1) cases with retinal lesions  Broad-spectrum antibiotic use: 15 (44.1) with retinal lesions. | - 19 of 34 patients (55.9%) with retinal lesions survived sepsis. - Multivariate analysis identified candidemia, infective endocarditis, broad-spectrum antibiotic use, and ocular symptoms as independent predictors of retinal lesions (all P < 0.05). |
| Simkiene et al,^32^ | NR | Management of Systemic shock: mechanical ventilation and noradrenaline  Specific interventions to manage the altered vessel architecture is beyond the scope of the present study. | - The study identified significant alterations in the retinal vasculature of septic patients - Increased central retinal arteriolar equivalent (CRAE) and - decreased vascular length density, suggesting microvascular changes associated with sepsis. |
| Huynh et al.^12^ | Positive blood culture | **Systemic**: antifungal therapy  **Ocular:**  with or without intravitreal antifungals | - About 16% of the patients developed ocular candidiasis. - No change in practice guidelines were advised though early detection for treatment was advised |
| Kirkegaard et al.^13^ | Positive blood culture | **Systemic**: Intravenous Fluconazole | - Cumulative rate of intraocular infection was 11.6% among patients. - Intraocular lesions were generally mild and did not require surgical intervention. - The median survival time was significantly longer in patients without eye involvement compared to those with ocular involvement. |
| Santanaraman et al.^14^ | NR | **Systemic:** Intravenous antibiotics  **Ocular** Intraocular antibiotics to all patients; 2 patients were advised vitrectomy, and one patient underwent lateral canthotomy | Two patients expired within a month of the infection. |
| Mohammad et al. ^19^ | - Positive blood culture - Visual complaints | **Systemic** antifungal therapy  Ocular: with systemic and intravitreal antifungal therapy. | Ocular examination identified 7 out of 161 (4.3%) patients with chorioretinitis or endophthalmitis. |
| Gluck et al. ^15^ | Positive blood culture | **Systemic** The duration of antifungal treatment was extended for one patient who was diagnosed with ocular candidiasis | Ocular candidiasis was rare among the study population, occurring in just 2.9% of those examined. |
| Yannis et al.^16^ | Positive blood culture | **Systemic**: Increased dosage of antifungals.  **Ocular**: 5 out of 7 patients received intravitreal Amphotericin B and one underwent vitrectomy. | - Chorioretinitis: 5.6% incidence rate. 57% of the patients with chorioretinitis were asymptomatic - Endophthalmitis: 1.6% incidence rate. |
| Ilhami et al.^22^ | NR | Diagnostic study only | - A significant association was found between the presence of Bacteremia-related retinal lesions (BRRLs) and higher Winston and APACHE II scores, - showing that patients with these lesions were more critically ill. - BRRLs were more in patients with central nervous system diseases or cancer. |
| Rodríguez-Adrián et al.^17^ | Routine evaluation after admission to ICU | Diagnostic only | - The study related the non-specific occurrence of retinal lesions in critically ill patients to systemic diseases than infections. - There was limited diagnostic utility of fundoscopy to find occult DBCI with underlying conditions |
| Chen et al. ^23^ | NR | **Ocular**: Patients received intravitreal injections containing vancomycin, amikacin; 6 patients underwent vitrectomy | - Klebsiella pneumonia association with liver abscess was consistent over 10 years. - Outcomes for the last 5-year review group were superior |
| Yaisawang et al.^24^ | - Positive cultures for melioidosis - raised blood titre with indirect hemagglutination (IHA) | **Ocular:** All patients had surgery. Most of the cases diagnosed as endophthalmitis and panophthalmitis required surgical intervention (five out of six cases, 83%), including pars plana vitrectomy (three out of five cases, 60%) and enucleation (two out of five cases, 40%). | - Orbital cellulitis was the most common   manifestation (seven cases, 44%);   - Only 5 (36%) patients had improved vision after treatment. |
| Ismail et al.^25^ | Reduced vision with eye pain and redness  Positive blood culture | **Systemic**: Intravenous antibiotics (cefepime, vancomycin, ceftazidime, ciprofloxacin).  **Ocular** intravitreal antibiotics (cefepime, vancomycin, ceftazidime, ciprofloxacin).  Due to worsening condition, a pars plana vitrectomy with silicone oil tamponade was performed to remove the sub-retinal abscess. | There was complete regression of intraocular inflammation and subretinal abscess. |
| Amir et al.^18^ | Loss of vision | **Systemic:** Hypotension was treated by stopping anti-hypertensive agents, salt tablets, midodrine, and encouraging oral fluid intake | Complete visual recovery |
| Krista et al.^26^ | Eye pain and redness | **Ocular:** The patient was treated with intravitreal injection of vancomycin (1 mg/0.1 cc) and ceftazidime (2.25 mg/0.1 cc), fortified vancomycin (25 mg/ml),  **Systemic** intravenous antibiotics | - After two weeks of antibiotics, the abscess and anterior chamber cellular infiltrate resolved. - The patient achieved a final visual acuity of 20/25 in the left eye. |
| Saidi et al. ^21^ | Presentation with preseptal cellulitis | **Systemic:** Intravenous cloxacillin, **Ocular:** Three intravitreal injections of vancomycin and ceftazidime.  Left eye underwent grid laser for diabetic macula edema | Resolution of vitritis and retinitis |
| Suchit et al^27^ | Pain, redness and reduced vision | **Systemic:** Intravenous ceftriaxone and **Ocular:** topical fortified cefazoline eyedrops.  Evisceration of the affected eye due to the progression of the infection | - The patient's condition did not improve significantly with antibiotic therapy - The left eye was ultimately eviscerated due to uncontrolled infection. |
| Allegrini et al.^28^ | Conjunctival chemosis, proptosis, ophthalmoplegia | **Systemic:** The patient was treated with intravenously administered antibiotics(piperacillin/tazobactam) and anticoagulation therapy with heparin. Then, oral antibiotics were given for one month. The affected teeth were extracted three weeks after the onset of ocular symptoms. | - After treatment, the patient no longer had ophthalmoplegia and periorbital edema. - Follow-up imaging showed normalization of the superior ophthalmic vein and periorbital fatty tissue. |
| Padmaja et al.^29^ | Diminution of vision | **Ocular:** choroidal drainage, three-port pars plana vitrectomy, drainage retinotomy, and silicone oil tamponade in both eyes.  **Systemic:** Additionally, intravenous amikacin and oral prednisolone were administered postoperatively. | - At 3 months follow-up, the patient's visual acuity improved to 20/160 and 20/630 with intraocular pressures of 11- and 6-mm Hg, respectively. - The choroidal detachment resolved, and the retina remained attached under silicone oil. - Subsequent cataract surgery further improved visual acuity to 20/100 in both eyes. |
| Karinya et al. ^30^ | Decreased vision | **Ocular:** The patient underwent three-port pars plana vitrectomy in both eyes to remove vitreous hemorrhages. Postoperatively, silicone oil was used for tamponade. Further surgeries were required to manage tractional retinal detachments and proliferative vitreoretinopathy (PVR).  **Systemic:** mechanical ventilation, hemofiltration | - The patient suffered significant visual impairment. Two years post-treatment, her vision was 6/36 in the right eye and counting fingers in the left eye, - Persistent macular holes and peripheral retinal detachments under silicone oil. |
| Chang et al.^31^ | Decreased vision | **Systemic**: intravenous cefotaxime and metronidazole  **Ocular**: Pars plana vitrectomy (PPV) with internal limiting membrane peeling by the inverted flap technique and gas tamponade | - Left eye BCVA 7 months later was 20/67. SD-OCT showed a closed MH with persisting photoreceptor layer disruption |
| Penelope et al.^20^ | Decreased vision | **Systemic**: amphotericin B and fluconazole  Amiodarone for atrial flutter  **Ocular**: Vitreous tap with intravitreal amphotericin B (5 μg), vancomycin (1 mg) and ceftazidime (2.2 mg) | - Despite aggressive treatment, both patients died due to overwhelming fungal septicemia |

Table Footnote: APACHE II: Acute Physiology and Chronic Health Evaluation II, BCVA: Best corrected visual acuity BRRLs: Bacteremia-related retinal lesions, CRAE: Central retinal arteriolar equivalent, DBCI: disseminated bacterial or candidal infections, IHA: indirect hemagglutination, MH: macular hole, SDOCT: spectral-domain optical coherence tomography NR: Not reported, PPV: Pars plana vitrectomy, PVR: proliferative vitreoretinopathy.
